# The “Cairo Accord”- towards the eradication of RHD: revisited (2026)

**DOI:** 10.3389/fcvm.2026.1799972

**Published:** 2026-05-08

**Authors:** Susy Kotit, Magdi Yacoub

**Affiliations:** 1 Aswan Heart Centre, Magdi Yacoub Heart Foundation, Aswan, Egypt; 2 Heart Science Centre, National Heart and Lung Institute, Imperial College London, London, United Kingdom

**Keywords:** Cairo Accord, disease burden, neglected diseases, rheumatic fever (RF), rheumatic heart disease (RHD), roadmap

## Abstract

Rheumatic heart disease (RHD) continues to be a significant global health burden, particularly in resource-limited regions of the world. This preventable and often neglected disease affects predominantly children and young adults, leading to considerable morbidity and mortality. In this article, we revisit the Cairo Accord that aims to address the challenges of RHD and set policy priorities for its eradication. We highlight the most remarkable progress in the field since the 2021 update, formulating updated recommendations. These include enhancing disease registries and echocardiographic screening, advance research on host and streptococcal strains genetics, biomarkers, vaccines, pharmaceuticals and penicillin prophylaxis, accelerate the development of regional Centers of Excellence, promoting MV repair and the development of tissue-engineered valve substitutes.

## Introduction

Rheumatic heart disease (RHD) remains a major health burden in resource-limited regions and among disadvantaged Indigenous populations in affluent countries (1–4). Women bear a disproportionately higher risk and burden of disease (5), leading to significant maternal and fetal risks during pregnancy (6–9).

Despite its significant contribution to mortality and disability, RHD continues to be neglected (10–12). While there is compelling evidence linking RHD to disadvantageous socio-economic (13, 14) and environmental conditions (15), the underlying mechanisms, especially factors influencing host susceptibility (16–21) and bacterial rheumatogenicity (22, 23), remain poorly understood. Addressing this issue effectively remains a high priority in public health (24, 25).

The “Cairo Accord on RHD”, formulated in 2017, set criteria and priorities to tackle the challenges associated with RHD towards its eradication. In this context, we revisit the topics outlined by the Cairo Accord (26) and its update (27) and discuss the progress made, incorporating insights from our own Aswan Rheumatic Heart Disease Registry (ARGI) (28).

### The “Cairo accord”: update on the recommendations

### 1. “Call for obtaining more accurate data on the epidemiology and natural history of the disease by strengthening the existing databases, and by ensuring that different databases are capable of cross-communication and data exchange.”

In recent years, efforts have focused on enhancing data collection and strengthening registries at global, national, and hospital levels to fill gaps in understanding RHD.

Global studies estimate the prevalence and mortality of RHD, revealing significant regional disparities. However, challenges remain regarding data quality and misclassification, particularly in low- and middle-income countries. National databases that use linked data have proven effective in estimating disease prevalence and characterizing patient populations. Despite this, there is still a notable absence of routine data on RHD, leading to a heavy reliance on national mortality records and hospital-based data.

### Global databases

The Global Burden of Disease Study (GBD) 2021 (29) by the Institute for Health Metrics and Evaluation (IHME) has enabled comprehensive research into the global burden (5, 30) and temporal trends (31–35) of RHD, attributable risk (31, 34), inequalities (36), and associations with socioeconomic development status worldwide (37).

**Figure 1:**
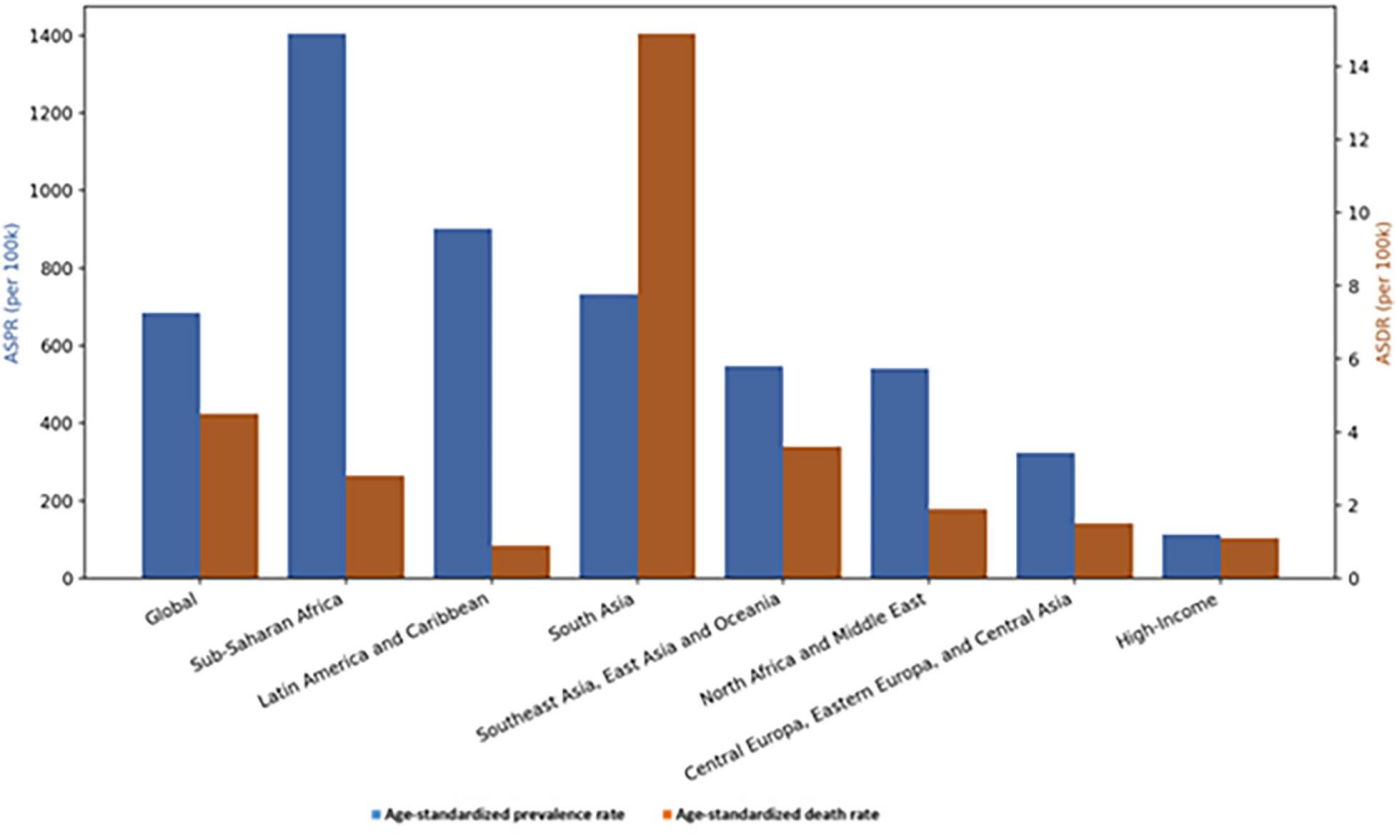
The distribution of age-standardized prevalence rate (ASPR) and age-standardized death rate (ASDR) at global and regional level for the year 2021.

In 2021, RHD affected an estimated 54.8 million people globally with an age-standardized prevalence of 684.2 per 100,000 causing over 373 000 deaths and 13.4 million DALYs (Figure 1) (38). The highest burden remains in Sub-Saharan Africa (15.5 million cases) and South Asia (14.4 million) (38).

**Figure 2:**
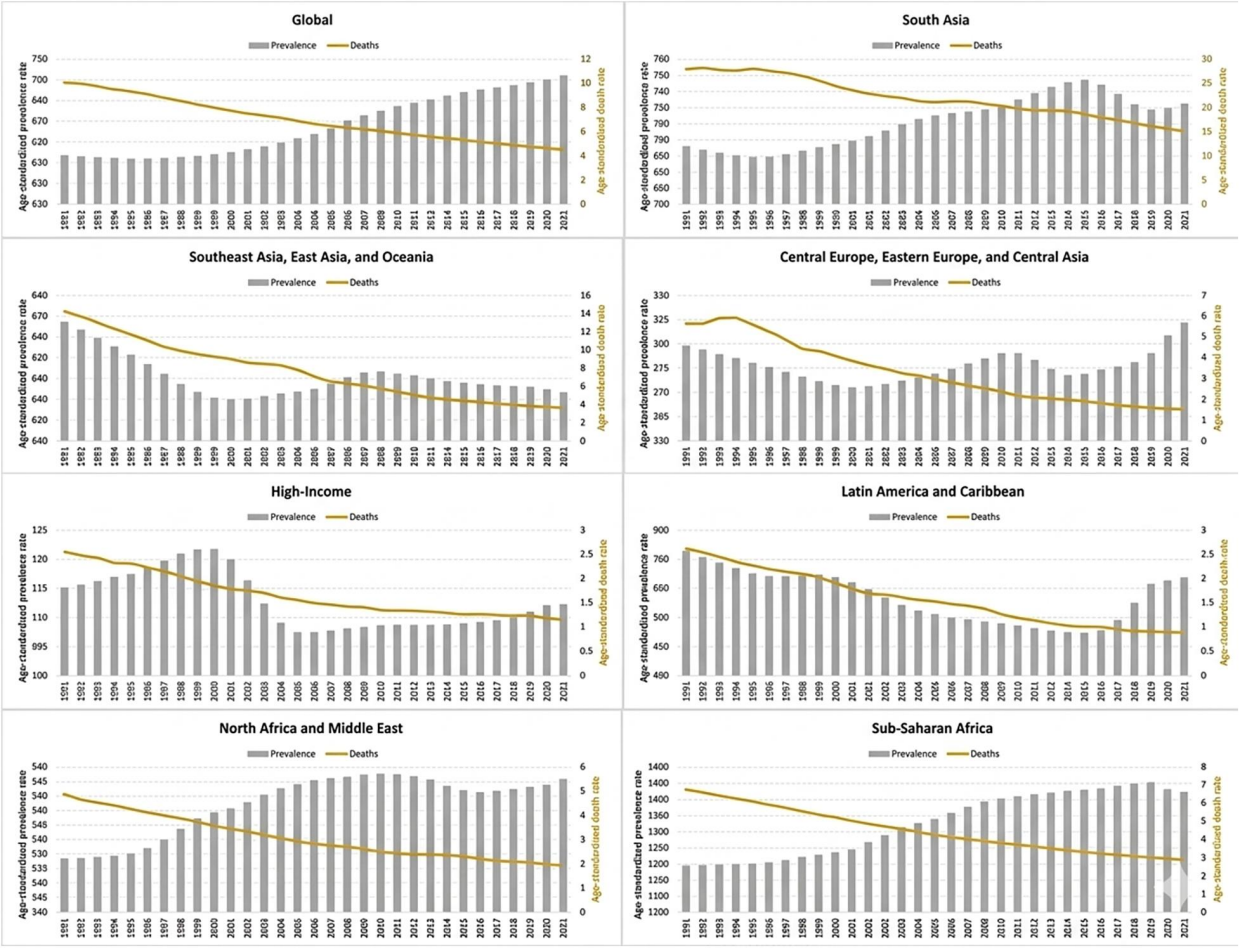
Trend of rheumatic heart disease age-standardized rates of prevalence and deaths at global and regional level between 1991 and 2021 [adapted (38)].

RHD disproportionately affects low- and middle-income countries (Figure 2) (33, 36, 39), especially children, adolescents, and women of childbearing age with girls aged 10–14 being particularly at risk (5). Accurate estimates are challenged by limited high-quality epidemiological data (38), particularly in sub-Saharan Africa.

Nonetheless, over half of the affluent nations in the European Union have witnessed a recent surge in RHD incidence rates across all genders. Potential factors driving this escalation encompass increased global migration from regions with higher RHD prevalence, and factors like housing and healthcare access for migrants (35). However, an analysis of RHD burden over 10 years in the USA identified that most recent cases of RHD are endemic and linked to socioeconomic deprivation (40).

From 1990 to 2019, both age-standardized prevalence (ASPR) and incidence rates (ASIR) of RHD increased, with projections indicating continued rises with ASPR anticipated to reach 559.88 per 100,000 population by 2030. The number of RHD-related heart failure cases is expected to reach nearly 3 million by 2030, with young people experiencing the highest prevalence and the elderly facing greater risk of heart failure and mortality. Females will remain the most affected with a rise in ASPR of RHD and RHD-linked HF (41).

Policymakers should use GBD data to guide cost-effective interventions and resource allocation (37), as RHD is projected to remain a persistent global health challenge through 2030 and beyond. Understanding disease trends and improving future projections are critical for effective control (38).

### National databases

National databases provide the most accurate estimates of ARF and RHD prevalence and burden (12) highlighting the underestimation of RHD in many high-resource settings and demonstrating the value of linked data (12) while improving understanding of disease progression and prognosis due to the longitudinal nature of the data. However, even after the successful implementation of these initiatives (12, 42, 43), the establishment of other national databases for ARF and RHD have been largely disappointing. This is mainly due to chronic underfunding, inconsistent standardized protocols for case definitions, screening, and data entry and logistical barriers such as poor healthcare access, absent specialized facilities, inadequate follow-up and weak integration into routine care, compounded by competing priorities like infectious diseases and nonrheumatic CVD (44), and shortages of echocardiographers and community health workers, all of which compromise case ascertainment.

Importantly, RHD Control Programmes have played a significant role in the cost-effective prevention and management of ARF and RHD in Australia. However, the number of ARF cases, influenced by improved awareness and surveillance, has increased showing the need for sustained investment and efforts, particularly for strengthening primordial and primary prevention efforts (45).

### Hospital based databases

Hospital-based registries play a crucial role in accurately and comprehensively collecting data on RHD, enabling the assessment of its burden, identification of best treatment practices, monitoring adherence to secondary prophylaxis, and evaluating treatment outcomes. Through this information, barriers to adherence can be identified to inform strategies and improve compliance. These registries have provided insights into the progressive deterioration of clinical outcomes in RHD, even among young patients, and demonstrated the benefits of therapeutic interventions (46).

Electronic patient registers are recommended to improve data access, patient management, and treatment adherence. However, establishing RHD registries in resource-limited settings is challenging due to underdeveloped healthcare systems.

### Aswan rheumatic heart disease registry (ARGI)

The ARGI, a prospective hospital-based registry at the Aswan Heart Centre, Egypt, collects comprehensive data on RHD patients nationwide (28). The registry encompasses various aspects of the disease, including demographics, mode of presentation, disease characteristics (clinical and echocardiographic characteristics, biomarkers, and genetics), treatment modalities, adherence to treatment, and disease outcomes. Regular follow-up monitors disease progression, the occurrence of adverse events and long-term disease outcomes.

The ARGI study, currently comprising over 2,595 patients, revealed poor outcomes and a 12.6% mortality rate despite the relatively young patient age (∼40 years) over an average follow-up of 53 months (28, 47). This registry provides crucial region-specific insights into the severity, progression, and phenotype of RHD, contributing to the understanding of regional epidemiology and supporting guided policy and efforts toward disease eradication, aligned with the Cairo Accord (27).

### International collaborations

Drawing from the ARGI model, a national RHD registry was established in Ethiopia, to address the local critical data gap and guide targeted prevention and management strategies (48). The registry prospectively enrolls all RHD patients across hospitals, clinics, and community programs, capturing demographics, ARF history, valvular pathology and severity, treatment adherence (penicillin and anticoagulation), and adverse events.

Implementation is proceeding in phases, staring with pilot cardiac centers, where healthcare workers received training in RHD diagnosis, treatment and patient enrollment, and referral networks were established to support subsequent national scale-up. The registry is designed to generate comprehensive, contemporary data on RHD burden to inform policy and resource allocation, identify progression risk factors for disease progression and adverse outcomes, and provide a platform for clinical and epidemiological research to reduce the burden of RHD (48).

### 2. “Conﬁne the use of echocardiographic screening programmes to research until further evidence regarding its impact on prognosis and cost-effectiveness is made available.”

Echocardiography is essential for accurately assessing the prevalence of RHD, particularly in community studies, offering insights into the full spectrum of the disease (49–53). Screening for undiagnosed RHD, especially in children, as they stand to benefit significantly from prevention of disease progression (54, 55), siblings and parents of ARF/RHD patients (56–59), young adults in high-burden, and socially vulnerable populations (60), holds promise for disease control (61). Focused screening during pregnancy in low- and middle-income countries is also critical due to risks to mother and fetus (9, 60, 62, 63), especially in the presence of mitral stenosis (62).

Recently, the World Heart Federation (WHF) updated its RHD diagnostic criteria (WHF 2012) (64), introducing a stage-based classification, a two-step echocardiography algorithm with screening and confirmatory criteria, management guidance, and recommendations for task-sharing with handheld devices (65). Designed for individuals aged aged 20 years and younger in high-prevalence, resource-limited settings, these updates enable broader active case-finding and early detection during screenings (65).

The 2023 WHF guidelines replace the 2012 discrete categories (borderline, definite, latent RHD) with a stage-based system (stages A–D) that stratifies patients by echocardiographic findings and risk of progression to advanced valvular disease, supporting monitoring, targeted management and secondary prophylaxis decisions.​

Most screening focuses on schoolchildren, underestimating the true burden since the disease correlates with poverty, and affected children may be less likely to attend school. Moreover, school-based studies might overlook the peak incidence of RHD which typically occurs after school age, with adults showing higher rates and more advanced disease (66). Screening young adults is therefore crucial.

The long-term outcomes of subclinical RHD remain unclear, highlighting the urgent need for longitudinal studies to investigate the natural history of latent RHD, identify clinical predictors of disease progression and develop simpler, universally applicable diagnostic criteria that can be applied across all healthcare settings (67).

### 3. “Enhance and coordinate research efforts on the genetics of rheumatogenic streptococcal strains and affected patients. The inﬂuence of ethnicity and epigenetics should be included in future studies.”

### Rheumatogenic streptococcal strains

**Figure 3:**
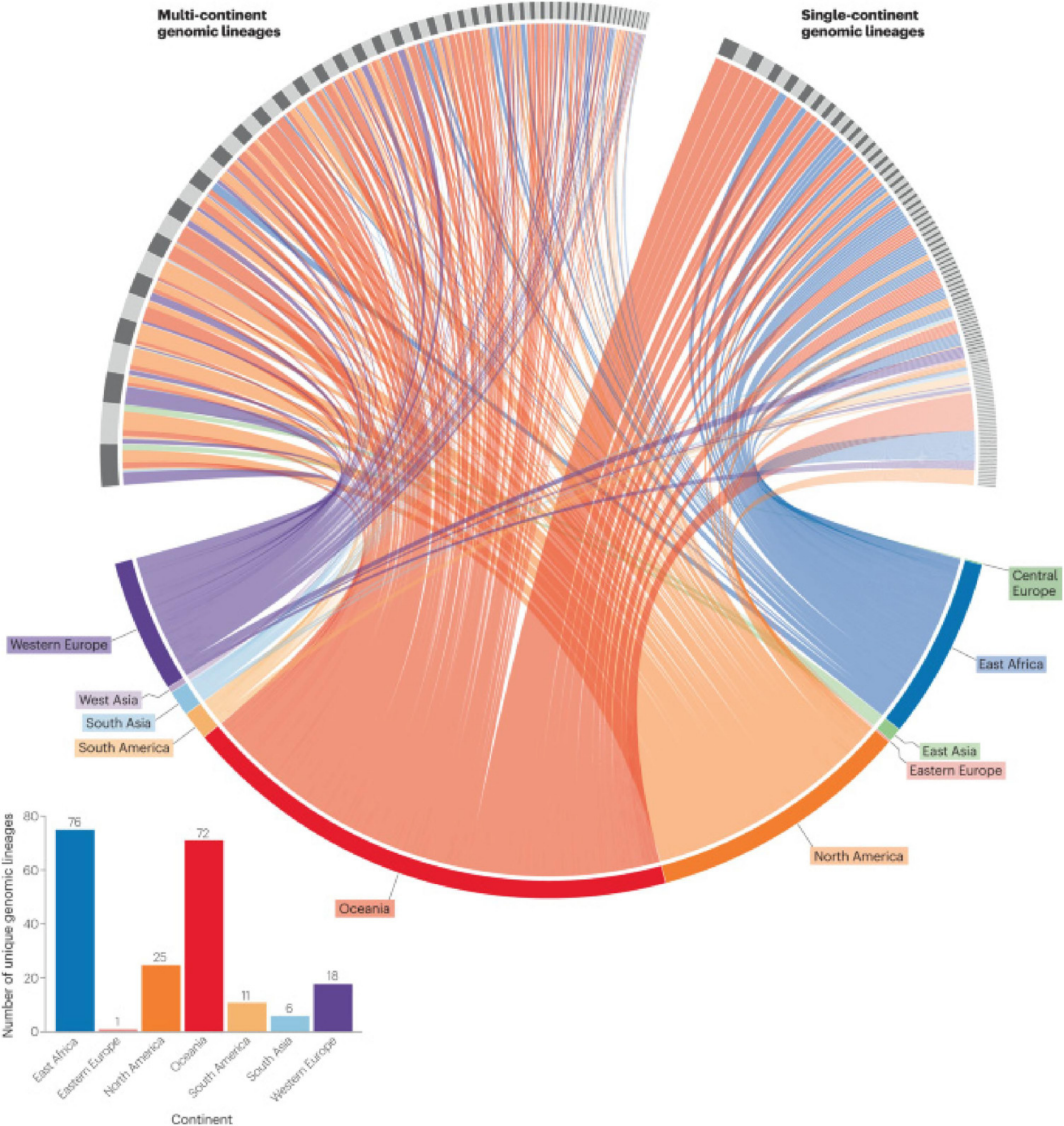
CIRCOS plot of 299 global group A *Streptococcus* (GAS) lineages based on 2,083 diverse GAS genome sequences (22). The connecting lines link genomic lineages with the geographical region of isolation. Colors relate to the geographical region of isolation. Genomic lineages are represented in the upper hemisphere of the plot (alternating grey) and split into lineages identified in multiple or single geographical regions. Bar plot represents the total number of “unique” single-continent genomic lineages per geographical region (coloured). (285).

Advancements in whole-genome sequencing have enabled the identification of various GAS populations and their associations with disease burden (68, 69). The distribution of GAS “lineages” or “lineages” varies geographically (Figure 3). Factors like transmission pathways, socioeconomic variables, and host-pathogen interactions influence the population structure (22, 70) resulting in a diversity of GAS emm-types (71). Recent studies have also shed light on the link between GAS skin infections and ARF (72–75) emphasizing the importance of strategies to prevent both GAS pharyngitis and skin infections (76).

In response to GAS infection, effector proteins like NAD-glycohydrolase (Nga), are delivered into host cells via streptolysin O (SLO), leading to the suppression of interleukin-8 secretion in macrophages (77). This highlights Nga and SLO as critical virulence factors associated with increased bacterial virulence (77). Additionally, streptococcal pyrogenic exotoxin B (SpeB) which cleave Gasdermin A (GSDMA) induce host cell pyroptosis (78, 79). Mucosal-associated invariant T cells (MAIT cells) also play a role in GAS infections (80).

The development of an effective vaccine against GAS requires a comprehensive understanding of its pathophysiology, global genetic diversity, transmission dynamics, and improved pathogen tracking. However, there is a notable lack of research on the genetics of rheumatogenic streptococcal strains, resulting in a critical gap in knowledge.

### Host genetic susceptibility

The involvement of host genetic factors in susceptibility to RF and its progression to RHD is suggested by the low RF rate, elevated familial incidence (56, 57) of RF and higher concordance rate in monozygotic twins compared to dizygotic twins (81). However, the contribution of hereditary vs. environmental risk factors remains uncertain (56).

A genome-wide association study (GWAS) in African individuals identified a susceptibility locus at 11q24.1, with an estimated polygenic heritability of 0.49 (82). In South Asia, GWAS revealed susceptibility signals in the HLA class III region (83), while HLA class II DR/DQ typing in North India showed significant differences in HLA DR∗ 15, HLA DR∗ B4, HLA DR∗ B5, and HLA DQB1∗ 02 between RHD cases and controls (81), implicating these regions in RHD susceptibility (83).

Associations have been found between specific single nucleotide polymorphisms (SNPs) such as TGF-*β*1 and IL-1β SNPs and RHD susceptibility (84), however, candidate gene studies in RF/RHD have been relatively small, lacking sufficient statistical power to establish robust and reproducible findings. Therefore, large-scale multicenter studies involving diverse populations are necessary to address this knowledge gap.

Proteomic analysis of plasma samples in African patients with severe RHD found elevated levels of adiponectin, complement C7, fibulin-1 (involved in valve tissue), and QSOX1 (a heart failure marker), indicating ongoing inflammation and valve damage. Conversely, lower levels of ficolin-3, important in microbial recognition, were observed. These findings suggest ongoing inflammation in RHD, even in the chronic phase of the disease, leading to progression in damage (85). Further research is needed to fully comprehend the role of these proteins as potential diagnostic or therapeutic targets for RHD as well as in assessing prognostic severity by recognizing varying degrees of inflammation (85).

The Genetics of Rheumatic Heart Disease Network (RHDGen) aims to identify genetic factors and biomarkers linked to RHD risk in Africans through a large case-control genetic association study, providing a platform for further study (86).

In women, estrogen amplifies immune responses and valve remodelling by driving B-cell activation, hypermutation, class-switch recombination, and pro-inflammatory T-cell responses, while downregulating the autoimmune regulator (AIRE) gene therefore, promoting autoreactive lymphocytes (87). These biological factors likely contribute to the female predominace in RHD, with women experiencing a 1.6–2 fold higher relative risk than men (88), despite RF affecting both sexes equally (89). Estrogen also promotes valve remodeling, fibrosis, neolymphangiogenesis, and hyaluronic acid synthesis (90, 91), accelerating mitral stenosis and regurgitation earlier in life.

RHD progression in females, is further driven by overexpressed X-chromosome immune genes such as CD40L, CXCR, OGT, FOXP3, TLR7, TLR8, IL2RG, BTK, and IL9R (92, 93) and prothymosin-alpha upregulation linked to estrogen receptor alpha (ER*α*), which boosts CD8+ T-cell cytotoxicity (92) and streptococcal antigen presentation (94).

Controlled Human Infection Models (CHIM) for GAS pharyngitis have allowed detailed study of early immune responses (95, 96). CHIM Volunteers showed elevated IL-1Ra, IL-6, IFN-*γ*, IP-10, and IL-18, with increase in monocyte and dendritic cells, reduction of B cells and CD4+ T cell subsets, activation of unconventional T cell populations (*γδ*TCR + V*δ*2+ T cells and MAIT cells) consistent with a robust early immune response important for host protection (96). In addition, symptomatic pharyngitis results in elevated C-reactive protein and inflammatory cytokines (interferon-*γ* and interleukin-6), and modest serological responses to streptolysin O and deoxyribonuclease B (95).

Immunohistochemical analysis of mitral and aortic valve tissue demonstrated marked infiltration of both CD4+ and CD8+ T lymphocytes throughout all valvular layers, with highest expression in the fibrosa, endothelium, and intravalvular microvessels, indicating active and vascular-involved inflammation. T-cell infiltration was more pronounced in mitral than in aortic valves and showed a trend toward greater intensity in females (97).

Histological and quantitative analysis revealed that females exhibit greater collagen deposition and glycosaminoglycan accumulation compared with males, indicating more pronounced fibrotic and extracellular matrix (ECM) remodelling, showing that gender shapes ECM dynamics and structural remodeling in RHD-affected valves (98).

These findings underscore the persistent immunopathological nature of RHD and point to gender-related differences in immune mechanisms driving chronic valvular injury, offering potential insights for the development of targeted immunomodulatory therapies.

### 4. “Enhance and coordinate global efforts to produce a vaccine. Strategies to accelerate the production of an effective vaccine (e.g., reverse vaccinology) should be explored and utilized.”

A mathematical model projects that introducing a Strep A vaccine between 2022 and 2034 could prevent 6 million cases of RHD globally (99). However, developing a universal vaccine against Group A Streptococcus (GAS) remains challenging due to the genetic diversity of GAS strains globally, the difficulty in identifying a universal protein that protects against all serotypes without triggering harmful immune cross-reactions (100–104), and the lack of substantial financial incentives for vaccine manufacturers.

M-protein-based candidates like StrepInCor, featuring a conserved 55-amino acid peptide, have shown specific antibody production without cardiac cross-reactivity and entered clinical trials by late 2023 (105–107).

The StreptAnova Phase I clinical trials have demonstrated the vaccine's safety and ability to generate functional antibodies capable of opsonophagocytic activity (95, 105, 108–110). Nevertheless, vaccine coverage varies geographically, as shown by a study in northwest Pretoria, South Africa (69), where 67% of prevalent emm types are not targeted by the vaccine (69), and only a quarter of skin infection isolates match the vaccine's antigens (111). Expanding global surveillance for circulating emm types and assessing cross-protection against strains are essential to improve vaccine coverage.

Other approaches combine synthetic peptides from M-protein epitopes and streptococcal virulence factors such as Spy-CEP (112–115), which show no cardiac or neurological risks in preclinical models (112, 114).

Non-M-protein candidates focus on the group A carbohydrate (GAC), a conserved GAS component essential for bacterial survival (116, 117) and virulence (116, 118, 119). Antibodies against GAC derivatives protect against GAS infection in animal models (105, 109). A novel conjugate vaccine combining SpyAD-GAC^PR^ with conserved virulence factors (C5a peptidase and SLO) elicits antibodies binding multiple GAS strains and protects mice from infection without cross-reactivity to human tissues. This supports integrating GAC into future multivalent vaccines for broad protection without autoimmunity (120).

**Figure 4:**
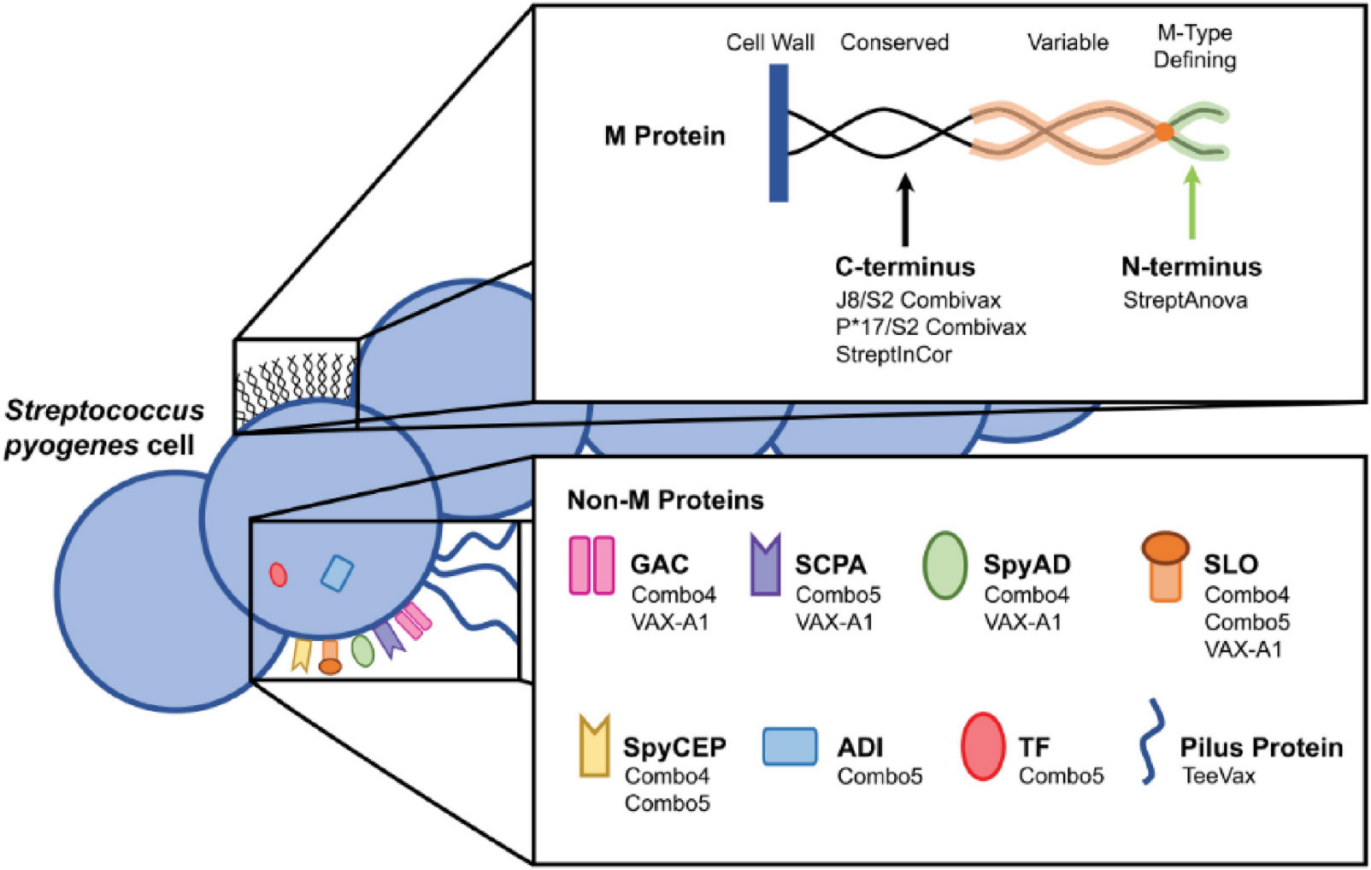
S. pyogenes antigens: Schematic of M protein and non-M protein antigens and corresponding vaccine candidates. (105).

Opsonophagocytosis assays reveal that multicomponent vaccines targeting diverse virulence factors enhance immune coverage, though combination immunizations may alter immune responses (120). Multicomponent vaccines can thus address concerns about limited vaccine coverage, though combination immunizations may influence the immune response (Figure 4) (120).

Inspired by mRNA COVID-19 vaccines, researchers at the University of Queensland and Moderna are developing mRNA formulations based on protein multi-component candidates (121).

To date, no Strep A vaccine candidate has obtained clinical approval or market availability. Accelerating vaccine development is critical, focusing on affordability for low- and middle-income countries and adopting innovative technologies such as bioconjugation and Generalized Modules for Membrane Antigens are critical to meeting global demand (122).

### 5. “Develop biomarkers for early diagnosis and follow-up of disease progression.”

The absence of a specific diagnostic test for acute rheumatic fever (ARF) hinders early detection, leading to underdiagnosis and high rates of RHD (123), especially in resource-limited settings. Differentiation between ARF from non-ARF cases and early ARF diagnosis is vital to start secondary prophylaxis with long-acting penicillin, preventing recurrent infections and heart valve damage. Currently, no immunologic or genetic markers reliably identify individuals at risk of RHD or predict disease progression.

Studies show that children with ARF have experienced approximately three times as many GAS infections as healthy peers living in similar environments (124), supporting the role of immune priming in disease development (71). ARF patients also exhibit broader antibody responses to GAS antigens, indicating progressive immune sensitization.

Preliminary transcriptomic analyses have identified candidate genes (CXCL8, TNF, CCR5) with distinct expression patterns in RHD, which may serve as biomarkers for disease progression (125). However, genome-wide differences between RF and RHD remain underexplored. Elevated neutrophil-to-lymphocyte ratio, neutrophil activation, and natural killer cell suppression appear important in both conditions. Changes in T cell function and cytokines such as IL-6, IFN-*γ*, TNF-α, IL-10, IL-12, and IL-17 influence RHD severity (126–128), though their reliability as markers requires further validation.

Gene polymorphism studies reveal higher cytokine levels (IL-4, IL-8, IL-1RA) and specific polymorphisms (IL2, IL4, IL6, IL10) in clinical RHD compared to latent cases, distinguishing disease stages (129). Similar cytokine patterns in RHD and chronic Chagas cardiomyopathy suggest shared inflammatory pathways, with IL-12p70, IL-1Ra, IL-4, and IL-7 effectively differentiating these diseases (128).

The IgG2 antibody response to N-acetyl-*β*-D-glucosamine (GlcNAc) differentiates ARF from uncomplicated pharyngitis and is linked to rheumatic carditis and chorea through molecular mimicry (130). IgG2 deposits on affected heart valves and neuronal cells highlight its role in autoimmune pathology, making it a promising biomarker (130).

Overall, these findings emphasize immune priming, dysregulated immune responses, and genetic factors in ARF and RHD development. They support exploring immune-modulating therapies and emphasize the need for further research to validate biomarkers and refine treatments to prevent chronic disease progression. Whole blood transcriptomics and large-scale omics studies are critical to fully understand systemic immune variations in ARF and RHD, and identify diagnostic and therapeutic targets.

### 6. “Provision of high-quality penicillin to affected areas—for both primary and secondary prevention—continues to be an important priority. In parallel, longitudinal studies that provide robust evidence for the beneﬁt of secondary prophylaxis on disease progression should be performed.”

Benzathine Penicillin G (BPG) is essential for preventing ARF recurrence, with the GOAL (Intramuscular vs. Enteral Penicillin Prophylaxis to Prevent Progression of RHD) trial showing that secondary antibiotic prophylaxis (SAP) significantly reduces RHD progression (131). However, intramuscular (IM) BPG requires high adherence (132, 133), and even minor lapses increase ARF recurrence (134) and subsequent heart valve damage. Unfortunately, global adherence to IM penicillin is below 50% (133) due to pain, travel, cost, and healthcare system challenges (132, 135–141).

While past efforts to improve adherence have shown limited success in enhancing adherence, even in well-resourced settings (132), oral penicillin may overcome many barriers, especially in low- and middle-income countries. Beliefs that IM penicillin is superior, are based on outdated studies using less effective oral formulations (142), leading some guidelines to support oral penicillin for RHD prophylaxis (143–145). The ongoing GOALIE (Intramuscular vs. Enteral Penicillin Prophylaxis to Prevent Progression of RHD) non-inferiority trial should provide information on the efficacy of intramuscular vs. oral penicillin SAP in preventing the progression of mild RHD (146).

Global BPG supply shortages further complicate RHD prevention (147), particularly in developing countries (148, 149). Ensuring reliable access to both IM and oral penicillin is crucial for effective secondary prevention.

In addition, optimal dosing frequency remains uncertain due to limited pharmacokinetic data in high-risk children and adolescents. Studies show that most high-risk patients on monthly BPG don't maintain protective penicillin levels, suggesting current regimens may be inadequate (150). Hollow Fiber Infection Models (HFIMs) and mathematical models indicate that higher unbound penicillin concentrations may be needed to prevent breakthrough infections, and that outcomes can vary by Strep A strain (151). Simultaneously, there is a need to refine the strategy of secondary prevention and RHD control.

Ideally, secondary prophylaxis would involve less frequent dosing, minimal discomfort through alternative delivery methods such as subcutaneous implants (152–154) and no cold chain. A phase 1 study showed subcutaneous high-dose BPG is safe, well-tolerated, and maintains protective levels for up to three months, potentially reducing clinic visits. However, challenges remain, including pain, skin changes, and large implant sizes. Continued research is exploring alternative subcutaneous infusion sites and implant size reduction (155, 156).

New long-acting penicillin formulations, including sustained-release implants, are in development but not yet clinically available. Further research is needed to optimize delivery, dosing, and patient acceptability, especially in resource-limited settings.

### 7. “Conduct studies that determine the potential value of anti-inﬂammatory/immunosuppressive therapy after acute rheumatic fever. Other important areas where evidence is needed include optimal stroke prevention strategies in patients with atrial ﬁbrillation and/or mitral stenosis, and pharmacological management of those with heart failure.”

Non-steroidal anti-inflammatory drugs (NSAIDs) effectively relieve ARF symptoms like arthritis (157, 158) but do not alter disease progression. Immunomodulatory treatments, including corticosteroids, have not shown clear benefits in improving cardiac outcomes in ARF patients (159–161) or preventing RHD (159), though corticosteroids may reduce inflammation in severe carditis (162–164). Further research is needed to assess their effects using modern imaging and immunological tools, and to explore combination therapies.

Hydroxychloroquine (HCQ) has shown promise in controlling inflammation in rheumatic carditis based on limited data (165, 166), but its efficacy remains unproven and requires more trials (166).

Studies reveal elevated complement factors and proinflammatory cytokines (IL-6, TNF*α*, IFN*γ*) in ARF and RHD (128, 129, 167, 168), suggesting potential for cytokine-blocking therapies like IL-6 blockade with tocilizumab (61).

Mucosal-associated invariant T (MAIT) cells are highly activated in Streptococcal Toxic Shock Syndrome (STSS) (80) and ARF (127, 169, 170), suggesting their contribution to disease pathology and represent targets for immunotherapy (171, 172), though research is ongoing.

Currently, there is no established effective immunomodulatory treatment to alter the long-term cardiac outcomes of ARF. Understanding ARF's immune mechanisms is critical to develop targeted therapies that impede chronic disease progression in ARF and reduce RHD burden.

### Heart failure

SGLT2 inhibitors (dapagliflozin, empagliflozin) significantly reduce cardiovascular death, heart failure hospitalizations, and all-cause mortality across heart failure patients regardless of ejection fraction, diabetes status, and concomitant treatment (173, 174). The favorable safety profile, minimal monitoring requirements, rapid onset of benefits, and positive impact on kidney function while improving quality of life make SGLT2 inhibitors the recommended therapy for heart failure (173, 174).

Iron deficiency affects up to 80% of acute heart failure patients regardless of ejection fraction. Meta-analysis [CONFIRM-HF (175), AFFIRM-AHF (176), and HEART-FI D^177^] shows that intravenous ferric carboxymaltose (FCM) reduces cardiovascular hospitalizations and death in iron-deficient patients with reduced or mildly reduced ejection fraction and should be considered standard care (178).

### Anticoagulation

Anticoagulation is a critical component of RHD management, substantially reducing the risk of thromboembolic events, stroke, valve-related complications in patients with atrial fibrillation, mitral valve stenosis, and prosthetic heart valves (179).

### Stroke prevention

Current guidelines emphasize vitamin K antagonists (VKAs), such as warfarin, as first-line therapy for RHD-associated AF and rheumatic mitral stenosis (mitral valve area ≤2.0 cm^2^) (180), as VKAs outperform rivaroxaban (NOAC) in preventing ischemic events and vascular mortality (181, 182), without increasing major bleeding (181, 182).

Oral anticoagulation in sinus rhythm is indicated after systemic embolism, with left atrial thrombus, or when transoesophageal echocardiography shows dense spontaneous echo contrast or a markedly enlarged left atrium (179).

### Mechanical prosthetic valves

Mechanical prosthetic valves require lifelong VKA anticoagulation. Safe anticoagulation for mechanical valve prostheses requires a time in therapeutic INR range (TTR) of 60%–70%, yet LMIC rates remain critically low at 20%–40% (183), with compliance failures in up to 92% of patients (184, 185).

Among RHD patients with mechanical valves, only 27% of INR tests are in range (183), with testing frequency plummeting from once every 3–4 weeks in the first year to <1 per year by year 7 (183).

Across Africa, 10% of patients discontinue warfarin entirely, 12% continue unmonitored (186), and 34% test sporadically (186, 187) with each 10% missed tests raising under-coagulation odds by 14% (188) directly precipitating valve thrombosis when INR <2 (189). Importantly, a significant number of patients is lost to follow-up (186),

Under-anticoagulation vastly outweighs over-anticoagulation risks (189), and mechanical valve thrombosis remains the predominant major valve-related event (190) in regions with poor INR control.

Preoperative atrial fibrillation affects 40% of RHD patients, with 20% developing it postoperatively (191), compounding prosthesis thrombosis and heart failure risks (192) in under-anticoagulation.

Pregnant women form a high-risk subgroup (193) due to pregnancy-induced hypercoagulability (194–196), while warfarin results in embryopathy and termination of pregnancy in the first trimester in over one third of pregnancies (195, 196).

Non-adherence predictors for anticoagulation in LMICs include young age (197), illiteracy (186), poverty (186), unemployment (196), female gender (197), low socioeconomic status (198), limited healthcare access (186), warfarin/INR testing unavailability, travel >1 h to clinics (185), forgetfulness (185), and misunderstanding therapy necessity (185).

Monitoring and management of anticoagulation pose serious challenges in resource-limited settings (186). Poor anticoagulation control remains the primary barrier to effective RHD surgical management in endemic regions, profoundly impacting mechanical valve outcomes and long-term survival.

To address this, warfarin clinics were established for the first time in Addis Ababa, Awassa, Gondar, and Bahir Dar through collaboration with the Cardiac Centre Ethiopia, Aswan Heart Centre and Chain of Hope. The clinics enroll all patients with AF or prosthetic valves without exclusion criteria, providing point-of-care INR testing, warfarin dose adjustment and provision, monitoring for complications, hospitalizations, and adverse events, rigorous compliance tracking, and comprehensive patient education. Regular follow-ups, standardized forms, and integrated registries ensure data continuity in this scalable, multidisciplinary model designed to improve outcomes in resource-limited settings (199).

### 8. “Accelerate the development of regional Centers of Excellence equipped with both physical and human resources to deal with prevention and treatment of the disease. Linking these centers into various regional and global research networks will signiﬁcantly enhance their performance and impact.”

There is a significant scarcity of region-specific data on the demographics, characteristics, disease progression, and long-term outcomes of RHD. This highlights the urgent need for comprehensive global efforts to better inform RHD policies and interventions.

The Aswan Heart Center (AHC), Magdi Yacoub Foundation, Egypt, is dedicated to high-quality cardiac care, with a strong focus on prevention, management, treatment, and research. The center also engages in community outreach, education, and research projects to advance cardiac health.

Within AHC, the RHD unit emphasizes holistic patient management, prevention programs, education, and long-term follow-up. The Aswan Rheumatic Heart Disease Registry (ARGI) is a key resource, providing valuable data on RHD prevalence, demographics, clinical features, and outcomes. ARGI supports trend analysis, evaluation of interventions, and continuous improvement in RHD care (28), aligning with the Cairo Accord's goals for eradication of RHD (27).

Similarly, the End Rheumatic Heart Disease Centre of Research Excellence (END RHD CRE) in Australia is focused on reducing ARF and RHD among Aboriginal and Torres Strait Islander Australians. Guided by the RHD Endgame Strategy, the initiative aims to eliminate RHD in Australia by 2031 through research (200), community engagement, and inclusion of patient perspectives (201). The END RHD CRE serves as a hub for researchers and collaborators addressing RHD across Australia (202, 203).

### 9. “Maximize the use of valve repair through educational programmes and exchange of expertise.”

As the progress in RHD prevention is insufficient, surgical intervention will remain essential for generations, yet it remains critically unavailable in many endemic regions (204–206). Global disparities profoundly impact RHD patients in LMICs, where over six billion people lack timely, safe, affordable care, with access ratios as low as one cardiac center per 33 million people in some endemic areas (204) covering less than 2% of the populations' needs (207).

In LMICs, RHD drives most surgical demands, for instance, 22% of patients aged ≥ 14 years require valve surgery within 30 months of diagnosis (208). Yet, less than 5% of eligible patients receive valve surgery or valvuloplasty within three years, even when symptomatic (209), due to resource shortages, late presentations, rural-urban divides, scarce valve repair expertise, intervention costs, and overburdened systems, leaving millions with untreated advanced valve disease.

Delayed or absent surgery elevates RHD mortality in low-income countries, mainly from heart failure or sudden death (209), with post-HF hospitalization mortality exceeding 40% at 30 days (209). Valve interventions markedly improve functional status and one-year complication-free survival (209, 210), cutting mortality risk by 77% (209).

Rheumatic valve surgery timing remains insufficiently addressed (211), in the absence of standalone global RHD surgical guidelines. However, early intervention is favored to prevent irreversible myocardial damage, especially in young patients (179, 212), also preserving repair feasibility before the development of advanced rheumatic changes (213).

Specialized RHD centers are urgently needed, where high case volume enhances successful, reproducible repairs, while refining indications and guiding policies. Robust postoperative infrastructure must support medical management, anticoagulation monitoring, access to follow-up and care, and quality-of-life evaluation.

In 2018, the Cape Town Declaration highlighted the dire lack of access to cardiac surgery for RHD in LMICs, urging an international coalition to endorse standardized cardiac care via clinical centers and specialized training (204). This led to the formation of the Cardiac Surgery Intersociety Alliance (CSIA), which prioritizes local capacity-building for pediatric and adult RHD surgery programs through government commitments, mentorship from RHD-experienced middle-income centers, and industry support. In 2020, Mozambique's Maputo Central Hospital and Rwanda's King Faisal Hospital Kigali became inaugural pilot sites with formalized mentorship and advanced training, advancing toward full independence (214, 215).

In Egypt, the Aswan Heart Centre continues to deliver structured training programs (216) and workshop series for surgeons, cardiologists, intensivists, perfusionists, scientists, and nurses through mentorship, capacity-building programs, and international networks to build regional cardiac surgery capacity in low-income settings.*Mitral valve repair*

Percutaneous mitral balloon commissurotomy (PMBC) is frequently used to aliviate rheumatic mitral stenosis (MS) when anatomically feasible. However, mitral valve repair (MVP) shows superior outcomes in restoring native valve function, particularly in young patients (217) with isolated mitral regurgitation (MR) or MS (218, 219), and pliable leaflets with minimal calcification (220, 221).

Repair results in improved left ventricular function and contractility (222), while avoiding lifelong anticoagulation risks (217, 223, 224), especially critical for girls to minimize pregnancy-related complications. MVP outperforms replacement in long-term survival (219, 225), morbidity, thromboembolic events (219, 222), irrespective of mechanical or bioprosthetic valves (219, 226), while mirroring reoperation rates at 10 years (227). Even post-PMBC, MVP remains viable in suitable morphology (228).

Outcomes favor mitral regurgitation (MR) over mitral stenosis (MS) (227) and decline in older patients with mixed valve disease or calcified valves (229), valvular fibrosis and scarring (225, 230), or concomitant aortic valve replacement (226). Careful patient selection, balancing reintervention risk while avoiding anticoagulation is essential.

A scoring model incorporating clinical and cardiac CT data can help predict early MVP success, guiding surgical decisions based on factors like calcification, leaflet morphology and papillary muscle symmetry (231).

Techniques for rheumatic MR repair encompass leaflet peeling to enhance pliability and length, cordal shortening or replacement, limited triangular resection or anterior free-edge augmentation of the anterior mitral leaflet, secondary cord release, and papillary muscle mobilization from the ventricular wall. A flexible posterior annuloplasty band is recommended for annular reduction and stabilization (213).

In rheumatic MS extended commissurotomy, commissural cord release, papillary muscle mobilization, and aggressive leaflet peeling are essential to restore pliability, length, and thickness. Thick chordae are addressed through thinning, fenestration, wedge resection, or replacement to optimize diastolic opening and minimize transvalvular gradients (218).

Secondary tricuspid regurgitation is common in RHD and demands concomitant intervention, with a low-threshold for tricuspid annuloplasty (232).

Although PMBC and MVP are preferred initial interventions in young patients with RHD, they often serve primarily to postpone inevitable valve replacement when anatomy deteriorates beyond repair (28, 233).

### Aortic valve repair

While rheumatic mitral valve repair yields encouraging outcomes, aortic valve repair remains challenging due to RHD's unique fibrosis, leaflet thickening, and commissural fusion (212, 218, 223). Nevertheless, AV repair can effectively address mild-to-moderate rheumatic aortic insufficiency and stenosis (234), with low hospital mortality and high 5-year survival (234).

Younger children particularly benefit, tolerating mild residual incompetence (223) while avoiding prosthesis-related reoperations and anticoagulation (223).

Early intervention, particularly during concomitant mitral surgery yields acceptable results in pliable early-stage tissue (223). However, AV repair is associated with high reoperation rates, often within 5 years (234), particularly in AV regurgitation (234).

Isolated aortic repair is rare, accounting for only 2% of cases (28, 235) owing to the multivalvular nature of RHD and predominant early mitral valve involvement (28).

Surgical techniques combining anterior leaflet peeling (decortication), chordal separation, focal decalcification (236, 237) enhance surface area, pliability and mobility, particularly at the commissures (223), outperforming simple shaving and yielding durable repair for aortic stenosis and regurgitation (238). Free-edge shaving/ decalcification offers no long-term durability advantage over bioprosthetic replacement (223).In rheumatic AR, autologous pericardial cusp augmentation with lozenge patch on the incised belly, preserving a native free edge, is superior to ribbon methods (223).

No ideal material exists for cusp augmentation/replacement, however, fresh pericardium is preferred for its softness and viability, despite suboptimal durability (213, 223). Tissue-engineered patches promise durable leaflet reconstruction and replacement in the future (239).

CT segmentation combined with 4D echocardiography mesh analysis, provide precise evaluation of valvular functional anatomy, pivotal for surgical planning to preserve left ventricular function (213). Prioritizing research on aortic root dynamics and tissue engineering will optimize much-needed valve repair.

Alas, given the nature of RHD, aortic valve repair remains limited, and therefore, surgical aortic valve replacement (AVR) remains indispensable for most patients.

### Valve replacement

In LMICs, most heart valve replacements occur in young patients with RHD (183). In Africa, over 90% receive mechanical valves (187) despite the benefits of bioprotheses (183), driven by fears of reoperation amid limited surgical capacity (207, 240). This however, leads to frequent redo surgeries for prosthesis thrombosis (241), as reliable anticoagulation proves challenging under socioeconomic constraints (183, 209), resulting in poor INR control and under-anticoagulation (183).

Valve thrombosis accounts for 74% of redo operations in LMICs, typically less than 4 years post-implantation, with 19% of redo patients needing recurring operations (241) and 73% presenting as clinical emergencies (242). Emergency reoperations carry a 3-fold higher mortality (190), and result in critical postoperative complications (241). Up to 25% of young patients (196) and 57% of women (183) with mechanical aortic valves for RHD suffer valve-related complications within 10 years, mainly in the form of major bleeding and thromboembolism (196). Pregnancy-induced hypercoagulability results in formation of thrombus on leaflets (195, 196), despite therapeutic anticoagulation (194). Mechanical valve thrombosis yields poor survival (190, 243, 244). Remote events likely cause underreported “valve-related” deaths, resulting in underestimation of true late mortality in LMICs (183, 245).

Women experience worse outcomes after AV and MV surgery in the form of higher in-hospital (246) and 30-day mortality (247), higher incidence of *de novo* dialysis, and prolonged stay in the intensive care unit, though long-term mortality is similar between genders (246).

Even in high-income countries, mechanical valves show a 2-fold higher mortality risk compared to bioprostheses inspite the latter's 2.8-fold greater reoperation rate (248–250), although, in children, bioprostheses achieve 92% 15-year survival vs. 76% for mechanical valves (251), with a reoperation rate of 59% compared to 68%, respectively.

Importantly, bioprothesis exhibit accelerated structural valve deterioration (SVD) in RHD patients (197) resulting in multiple reoperations in young patients (252), with comorbidities like left ventricular dysfunction, pulmonary hypertension and prior sternotomies, elevating redo risks (252).

Reoperation mortality favors aortic and mitral bioprosthesis over mechanical valves (253–256), warranting critical reappraisal of selection criteria especially given evidence gaps in balancing mechanical valve anticoagulation risks against bioprostheses reoperation burden in young LMIC RHD patients. Decellularized aortic homografts (DAH) offer promising aortic valve replacement alternatioves, with excellent 5-year haemodynamics and freedom from death, reoperation, endocarditis, bleeding, and thromboembolism (257).

Emerging flexible leaflet polymeric heart valves (PHVs) (258) address current prostheses limitations through optimal hemodynamics, biocompatibility, low thrombogenicity, longevity, transcatheter potential, and global accessibility (258, 259).

### Ross procedure

The Ross procedure (pulmonary autograft AVR with RVOT homograft) in RHD achieves equivalent 20-year outcomes of survival, structural valve dysfunction (SVD), reoperation rates and valve-related events (stroke, TIA, cerebral hemorrhagic event, IE) compared to non-RHD aetiologies, despite RHD's baseline LV dysfunction, heart failure, and multivalve involvement (260). Meticulously placing the autograft in an intra-annular position to fully support its valve cusps, while adressing aortic annulus dilatation (260) is essential to prevent or dalay dilatation of the neoaortic root.

Pulmonary autograft dysfunction due to RHD is uncommon, however, adequate prophylaxis is advised, especially in patients under the age of 30 years (260). A key advantage of the Ross procedure is the abdence of lifelong anticoagulation requirements. A major limitation for the procedure is the availability of homografts.

### Transcatheter procedures

In inoperable patients with multivalvular RHD, transcatheter procedures may be feasible even in the presence of a previous mitral mechanical prosthesis (261).

### Transcatheter edge-to-edge repair (TEER)

Transcatheter edge-to-edge repair (TEER) (262), offers a less invasive option for for rheumatic MR in high-surgical-risk patients (263), despite challenges from RHD's hallmark leaflet thickening, calcification, and restricted mobility complicating grasp and coaptation (263, 264).

TEER yields higher hospital complications, crossover to surgery, and 90-day readmissions (263), for RHD but comparable in-hospital mortality, confirming feasibility in select patients with pliable leaflets, minimal calcification, and no alternative therapeutic option (263).

### Transcathether aortic valve replacement (TAVR)

Originally for degenerative calcific stenosis, TAVR shows expanding utility in rheumatic aortic stenosis (RAS), with better 1-year prognosis, despite higher-risk profiles in the presence of elevated NT-proBNP, STS scores, atrial fibrillation, cardiac enlargement, valve regurgitation, reduced ejection fraction, and lower eGFR (265).

TAVR also mirrors surgical aortic valve replacement (SAVR) mortality rates while reducing complications and redo interventions, with the exception of higher permanent pacemaker rates (265, 266).

Challenges owing to rheumatic leaflets’ fibrosis, restricted mobility, and late calcification (266, 267) elevate deployment risks (265), yet TAVR proves safe and feasible for RAS (265), offering a critical lifeline for inoperable LMIC patients lacking alternatives.

In LMICs, RHD patients are young and present late with pure aortic regurgitation (AR) and severe ventricular remodeling (259), spurring interest in extending TAVR indications to rheumatic aortic regurgitation, which has shown a 3-year mortality equivalent to non-rheumatic AR, in addition to lower paravalvular leak rates (268).

Current TAVR limitations such as limited leaflet durability, high costs, and secondary procedure requirements, restrict its use to elderly patients in high-income countries.

The SAT balloon-expandable TAVR system addresses RHD-specific requirements through pacing-free, non-occlusive, minimal-oversize deployment (259, 269). Key innovations include shape-stabilized scallops with plastic deformation anchoring in non-calcified annuli (259, 269), an hourglass-shaped stent sparing the conduction system (259, 269), spacious neo-sinuses reducing subclinical leaflet thrombosis (269, 270), and non-thrombogenic polymer leaflets enhancing longevity for young RHD patients (259).

Prospective studies validating long-term outcomes and durability are essential.

Industry must prioritize underserved LMICs, tailoring TAVR systems for this vulnerable population by enhancing bioprosthetic longevity through decellularized tissues and non-degrading polymer leaflets (259), avoiding cost barriers, rapid pacing and risks of permanent pacemaker implantation (259).

### Transcatheter tricuspid valve replacement (TTVR)

Transcatheter tricuspid valve replacement (TTVR) offers a feasible alternative in select patients with high surgical risk (271, 272). However, RHD patients are typically excluded from TTVR trials (273) due to concerns over valve durability, asymmetric leaflet pathology, and confounding multivalvular disease (273).

Given tricuspid involvement in the majority of the RHD cases and the risks of isolated tricuspid valve surgical reintervention, expanding TTVR indications could provide lifesaving, less invasive access for standalone tricuspid disease.

### Valve-in-valve (ViV)

Valve-in-valve (ViV) shows promise as a redo surgery alternative for RHD patients with failing bioprostheses (252), offering equivalent device success, good mid-term outcomes over a median 20-month follow-up, fewer major adverse events, shorter stays, and preserved future access for surgical or transcatheter interventions. The elevated 30-day mortality reflects RHD's higher comorbidity burden and the presence of multiple reoperations (252).

Technical challenges include small annular size, coronary access issues, and limited RHD-specific long-term data (252). ViV suits select high-risk patients, but large prospective studies defining outcomes and optimal access routes remain essential (252).

### 10. “Develop tissue-engineered valve substitutes, including percutaneous valves that are both affordable and simple to implant.”

Current heart valve prostheses do not replicate the intricate function of natural heart valves. Next-generation replacements using tissue engineering are needed. Valves that can self-repair, adapt to cardiovascular changes, avoid immune reactions, and eliminate the need for lifelong anti-thrombotic therapy will provide a long-lasting solution for patients, especially benefiting children and young adults.

Recent studies using aptamer-immobilized decellularized porcine aortic valves (aptamer-DAVs) have shown enhanced scaffold endothelialization by promoting cell adhesion and growth, though their mechanical properties require further evaluation (274).

Another approach involves living tissue-engineered heart valves (Living-TEHVs), created by repopulating decellularized scaffolds with patient-derived stem cells, such as fibroblasts and endothelial cells from adipose tissue. Preconditioned in bioreactors, these valves demonstrate promising hemodynamic performance and aim to mimic native valve mechanics (275). Further research is needed to optimize stem cell sources, seeding techniques, long-term outcomes, and comparisons with cell-free scaffolds (276, 277).

**Figure 5:**
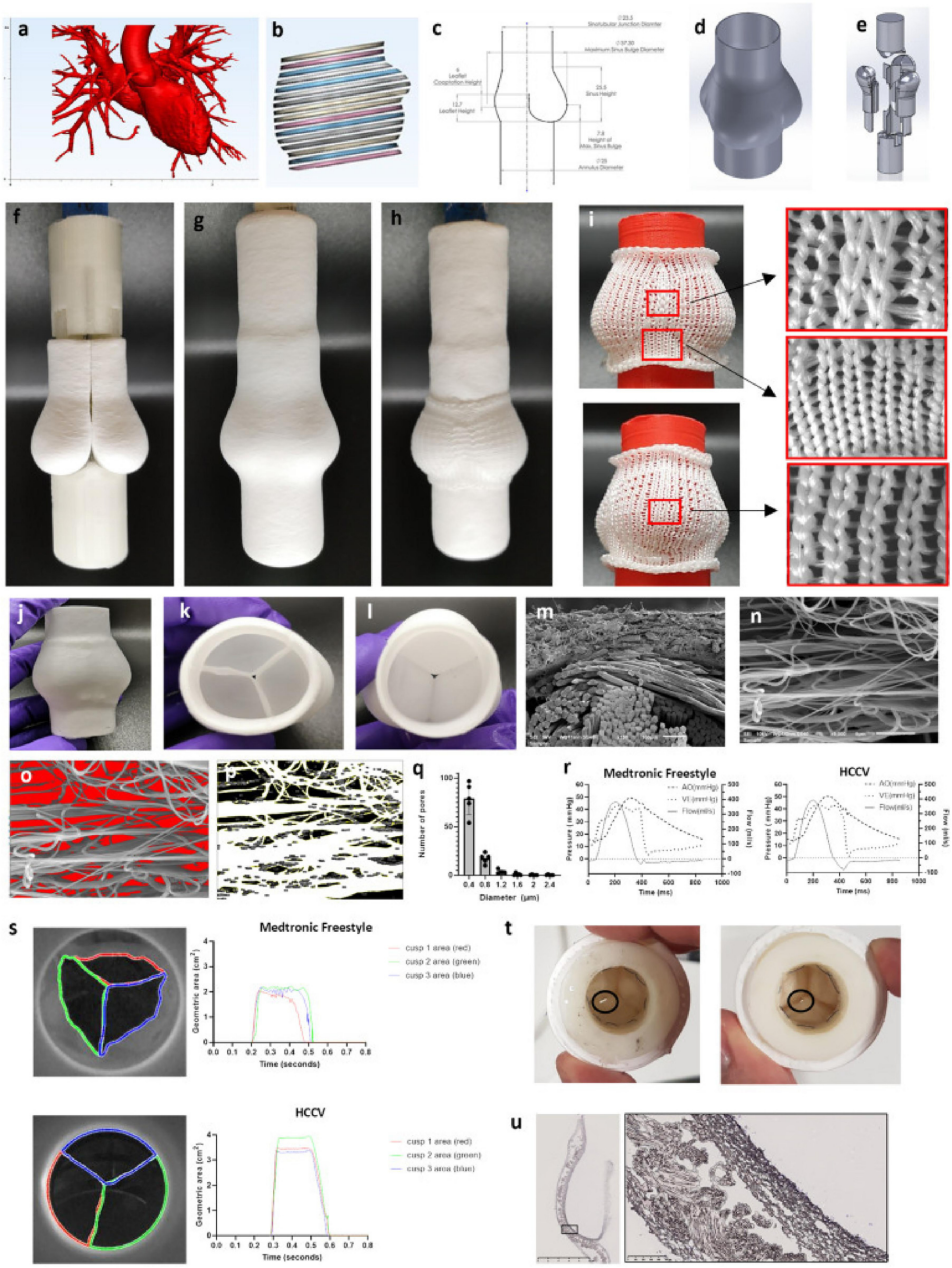
A CT scanned image of a normal aortic root used as a template for designing the HCCV a; segmentation of the root region b; 2D drawing of the components of the HCCV (all units are in mm) c; 3D computer-assisted design (CAD) of the external geometry d; valve assembly where one-third of the HCCV was modelled to the shape of a single sinus, leaflet and one third of ascending artery and 3 of these sinus models were assembled to create the complete HCCV e. A holder was designed to house the 3 sinus models and to create the ascending artery. A counter mould was designed to complementarily fit the sinuses to create the interleaflet triangles and the annulus e. Three jet sprayed sinus moulds and housed into the holder (top) with the counter mould (bottom) f. Whole mould assembly jet sprayed g with the knitted support h. Pictures showing the design of the knitted support with 2 sinus view (top) and one sinus view (bottom) with regions of magnification of the different regions i. Final HCCV after the dissolution of the PVA moulds j showing top view k and annular view l. SEM through the wall of the sinus showing the knitted PCL support (bottom fibres) and nanofibers (top) × 150 m. SEM of nanofibers ×5,000 n; analysis of the empty spaces between the nanofibers o, threshold image p, and a histogram of pore size distribution [all data is represented as mean (±SD), *n* = 5, independent samples] q. Representative pressure-flow relationships of the Medtronic Freestyle valve and the HCCV r. Representative geometric areas of the 3 leaflets s. Each leaflet's opening area was assigned 3 different colours to show their synchronisation between the Medtronic Freestyle valve (top, *n* = 1) and the HCCV (bottom, *n* = 1) s. Tears (indicated by black circle) in 2 HCCVs after 9 million cycles in the accelerated wear tester t. Cross-section of the HCCV with high magnification of the sinus wall showing the knitted support embedded within nanofibers u. (278).

Recent advances include the Heart Biotech Composite Component Valve (HCCV), a novel anatomically precise, acellular, synthetic pulmonary valve scaffold designed to mimic native valve geometry and function (Figure 5). The HCCV has shown favorable hydrodynamic performance *in vitro*, promising durability and *in vivo* regenerative capacity, with *in situ* valvulogenesis, integrating relevant cell types, extracellular matrix, and regulatory components, marking a significant advance toward living, regenerative heart valve replacements (278).

**Figure 6:**
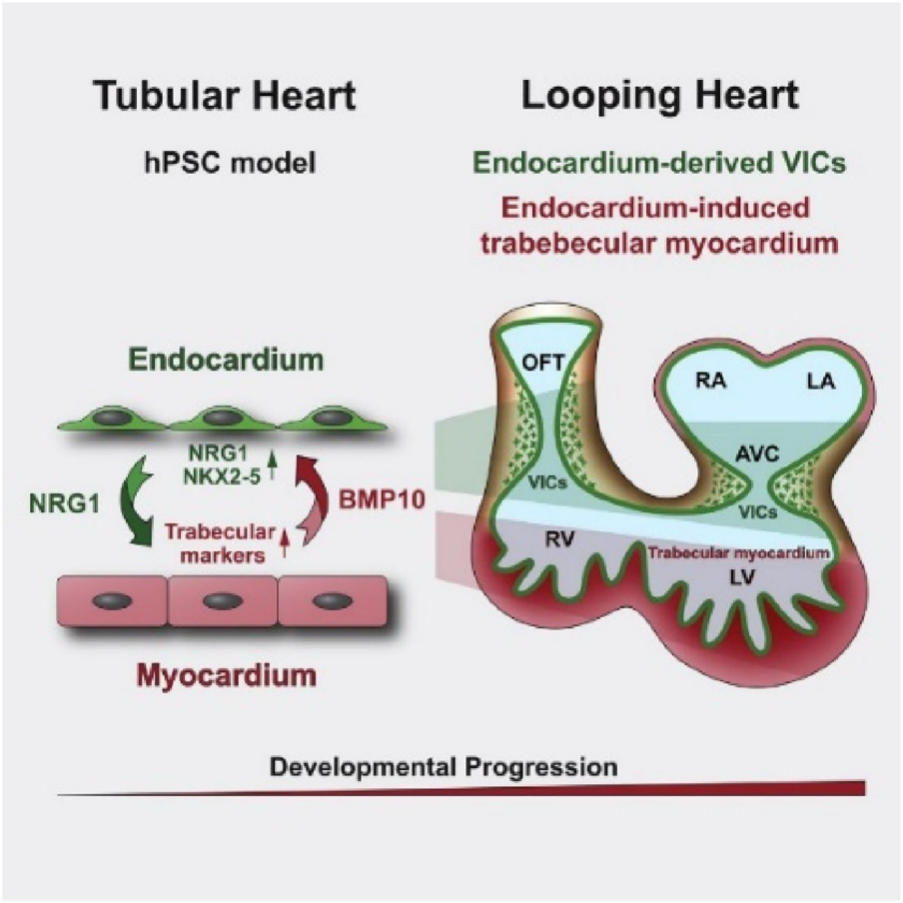
BMP10 signaling promotes the development of endocardial cells from human pluripotent stem cell-derived cardiovascular progenitors (279).

The identification of BMP10 as a key regulator of endocardial development highlights the value of human pluripotent stem cell (hPSC) models in understanding heart cell differentiation (Figure 6). These models facilitate the design of engineered heart tissues that replicate native cellular composition, advancing studies in heart development, disease, drug testing, and providing sources of vascular endothelial and valve interstitial cells for regenerative therapies (279).

## Discussion

RHD remains a major global health challenge (31–34), particularly in resource-limited regions, with its true burden often underestimated due to poor data and misclassified mortality. Despite progress since the Cairo Accord, greater efforts are needed to raise the global priority of acute rheumatic fever (ARF) and RHD.

Comprehensive analyses of region-specific data on demographics, disease features, and outcomes collection through robust registries at global, national, and hospital levels are needed to fill knowledge gaps, understand disease progression, address disparities, inform policies and tailor interventions. Long-term studies are needed to clarify the natural history of latent RHD, identify clinical predictors, and determine the role of screening. Universal, simplified diagnostic criteria are also critical.

Genetic research reveals the complexity of RHD susceptibility, emphasizing the need to identify susceptibility loci (82, 83). Early diagnosis and improved biomarkers will enhance prognosis, and anti-inflammatory and immunomodulatory therapies hold therapeutic promise. Further evidence on the efficacy of secondary antibiotic prophylaxis (SAP) is needed, with better knowledge of penicillin pharmacokinetics and alternatives, including implants, to optimize therapeutic strategies and regimens.

Developing polyvalent streptococcal vaccines requires substantial funding and a deeper understanding of Group A Streptococcus (GAS) genetics, distribution, transmission, and host interactions (22, 70).

No universally perfect repair method exists for rheumatic valve disease, emphasizing the importance of tailored approaches guided by patient profile, cardiac status, and valve pathology, leveraging multimodality imaging for informed decision-making. In young, active patients from endemic areas with limited healthcare access, aggressive valve preservation, even temporarily, is warranted. Bioprostheses offer good long-term outcomes while avoiding anticoagulation. Evolving valve-conserving techniques, polymeric and tissue-engineered valves and transcatheter alternatives require validation through large-scale, long-term multicenter studies comparing durability and outcomes, while refining indications and guiding policies. Specialized RHD Centers of Excellence with high case volumes are urgently needed to ensure reproducible repairs, supported by robust postoperative infrastructure and anticoagulation monitoring. Regional capacity-building through surgical exchange programs, partnerships, mentoring, and training will drive research-guided prevention, management, and treatment. Pregnant women with RHD face heightened risk of adverse cardiovascular events (9). Risk-stratefied cardio-obstetric care is essential for counselling, anticoagulation optimization, and treatment to prevent maternal and fetal adverse outcomes (280).

Addressing socioeconomic factors and improving access to prevention and care are crucial to reducing disparities. Integrating RHD into global aid frameworks, especially in underprivileged nations, is vital to prevent future increases in disease burden (30).

Implementing prevention, effective heart failure management, and valve surgery could significantly reduce mortality in Africa by 2030 (281). Achieving this requires a comprehensive ARF/RHD roadmap at at global, national, and local levels, focused on cost-effective control (282). Despite growing recognition of RHD as a global health issue, research and funding remain inadequate compared to other neglected diseases (283, 284). Urgent efforts matching the true burden of disease are needed for prevention, early detection, and the development of a safe GAS vaccine.

## Conclusion

Progress has been made since our update on the Cairo Accord, yet much remains to be done to raise the global priority of ARF and RHD.

Improving data, prevention, diagnostics, genetics research, access to care, intervention expertise, and international collaboration requires sustained funding, training, and policy support.

Persistent, comprehensive action as outlined in the Cairo Accord is essential to eradicate RHD and reduce its global burden.
